# The General and Special Pathology and Therapeutics of Dr. J. L. Schönlein, Professor in Zürich

**Published:** 1846-01

**Authors:** 


### ART. II.

*Dr. J. L. Schönlein's, Professor in Zürich, Allgemeine und Specielle Pathologie und Therapie, &c. &c.* In Vier Theilen. *St. Gallen,* 1839.

*The General and Special Pathology and Therapeutics of Dr. J. L. Schönlein,* Professor at Zürich. *Edited by several of his Class, from Notes taken at his Lectures.* In Four Parts. The Fourth Edition, much-improved. *St. Gall,* 1839. 8vo. pp. 327, 276, 281, 208.

*Schönlein's Klinische Vorträge in dem Charité Krankenhause zu Berlin.* Redigirt und herausgegeben von Dr. L. GÜTERBOCK. Erster und Zweiter Heft, 1842; Dritter Heft, 1844. *Berlin.*

*Dr. Schönlein's Clinical Discourses in the Charité Hospital at Berlin.* Taken and edited by Dr. L. GÜTERBOCK. In Three Parts. 8vo, pp. 480. *Berlin,* 1842. 1844.

*Dr. Schönlein als Artz und klinischer Lehrer aus der Schilderung der Dr. Güterbock; einer unabweisbaren Kritik unterworfen* von Dr. LEHRS und Dr. SCHARLAU. *Berlin,* 1842.

*An unimpeachable Critique on Dr. Schönlein, as Physician and Clinical Lecturer, &c. &c.* By Dr. LEHRS and Dr. SCHARLAU. 8vo, pp. 201.

WE feel that an apology is due to our readers for our tardy notice of the pathology and therapeutics of Schönlein. His fame is European, and whether justly or unjustly awarded, that extended reputation was reason sufficient for his trial in our critical crucible. If, however, we were to print a whole page of excuses our readers would be little the wiser; therefore, we content ourselves with observing that the delay has been advantageous, inasmuch as it enables us to judge of the clinical performances of Schönlein, as well as of his labours at the desk. We begin with the latter.

Medicine concerns itself with life generally, and with the life of man specially. Man is a part of the general system of the universe, and in common with all other organisms, seeks to separate himself from the great whole, and to be manifested as an independent entity. The great whole resists, antagonizes this effort;—a contest arises between the egoistic and planetary principles; and so long as the latter is equipoised or overcome by the former, there is health to the individual. The ascendancy of the planetary principle is death. Diseases is the struggle of the egoistic with the planetary—the resistance of the individual organism against injurious agents (schädlichen potenz) which seek to destroy it. These struggles appear under diverse forms which mark the forms of disease. The form of disease may be determined by three circumstances: 1. The nature of the hurtful agents. 2. The organs on which the hurtful agents act. 3. The individuality of the subjects in which diseases are developed. Hurtful agents are divided into two classes, accordingly as they are of internal and external origin. The external may be, *a,* Alimentary, and act on the chylopoietic viscera. *b,* Atmospheric, and act on the lungs and skin. *c,* Cosmic, operating on the nervous system. *d,* Chemical, which appear sometimes as injurious agents, sometimes as curative agents: poisons comprised in the last division may be arranged in two classes, the one being represented by arsenic, the other by prussic acid. *e,* Mechanical agents: these differ according to their mode of operation and the organ on which they act. Stone in the bladder or gall-ducts, and monstrosities belong to this head. Hurtful agencies of internal origin may consist, *a,* of increased or diminished power, physical or psychical, as paralysis of the muscles, loss of memory, &c.: *b,* in suppressed or increased secretion, as of the saliva, urine, &c.: *c,* in a peculiar derangement of the soul, with which insensibility to other impressions is connected. The emotions belong to this class, which may be divided into the depressing and exciting.

The second circumstance determining the forms of disease is the organ on which the hurtful agent acts. All organs have a receptivity for hurtful agents,—a predisposition to disease, which is greater or less as the organ is, or is not, in a state of evolution. The evolution may be either permanent or periodic; the former may be subdivided into the true period of evolution, or youth; middle age; and the period of involution, or old age. The physiological characters of these successive stages of development and decline are known, as well as their pathological relations, or (in Schönlein phrase) the receptivity of the organs during each stage.

The third circumstance determining the forms of disease, is the individuality of the subject on which the hurtful agencies operate. The points to be noticed specially under this head, are, the temperament, sex, and idiosyncracy.

*Contagion.* Schönlein breaks off abruptly from the true sequence of his ideas to the subjects of contagion and spontaneous generation. We, however, follow the master mediciner in his own way. There are hurtful agencies which originate in the organism, and excite disease, and which are communicable to another organism, and excite similar disease. They are developed from spontaneous diseases, either in solitary or many individuals. The contagions developed in solitary individuals have their origin in the so-called acrid humours (Schärfe, acrimonia.) Acrimony of the humours is understood to mean a peculiar derangement of the quality of the animal chemistry: as, for example, a peculiar condition of the blood, saliva, &c. Two particulars are to be noted regarding contagion: 1, the basis, or materies; 2, the ætherial or active principle. The materies may be in the form of a fluid, a vapour, or a gas. The nature of the active principle is unknown, but Schönlein thinks it allied to free electricity; because firstly, it is perceptible to the taste and smell, as is also galvanic action (!); secondly, because certain electrical bodies are the best media of contagion, as glass, resin, silk (!).

*Symptoms of disease.* Every hurtful agent acts only on its own proper organs: as, for example, the atmosphere on the lungs: consequently it excites only a local disease, and not a general affection in the widest sense. The egoistic principle, however, reacts against the injurious agent, and a series of phenomena are developed, which were not manifest in the healthy organism. These phenomena are the symptoms of disease. There is, however, no true signum pathognomicum: dread of water, for example, is present in the more intense forms of typhus as well as of hydrophobia; but there are symptomata symptomatum. Symptoms are subject-objective, when they are expressed by the patient; object-subjective, when noted by the observer. They also may be symptoms of the cause of disease arising directly from the operation of the injurious agent; or symptoms of the disease, and originate in the reaction of the egoistic principle. If the hurtful agent is not intense, or if the organ be unimportant, the symptoms are local; if the contrary, the whole organism is implicated. According to Schönlein, this sympathy of the organism with the local affection constitutes fever.

*Fever.* Fever is rather the shadow of the disease than the disease itself. The mode of reaction of the egoistic principle is threefold. 1. It is sufficiently powerful to remove at once the injurious agent, and maintain the integrity of the system: this is erethismus. 2. The reaction is greater, and the struggle to remove the injurious agent more prolonged: this is synocha. 3. The reaction is too weak: this is torpor. If, for example, food acts injuriously on the alimentary canal, reaction of the egoistic principle takes place, vomiting follows, and the injurious agent is removed. Should the phenomena not cease here, but vomiting continue, and sensibility and irritability be developed, the reaction is too strong, and takes on the character of synocha; but if vomiting do not supervene, the reaction is too weak, and it assumes the torpid form. Just then as reaction is threefold, every topical affection and every form of fever is threefold. Erethism is the centre, synocha and torpor the two poles. The stage of convalescence from either of the poles must be through the erethistic. If it go beyond this, paralysis (lähmung) takes place: the organs affected cease to perform their functions. This paralysis or lameness may affect the nervous system, when it induces paralysis properly so called; or it may affect the vascular system, and gangrene be developed. In every disease, whether local or general, the nervous and vascular systems are active. The affection of the former is shown by the subjective symptom pain: of the latter, by the objective symptoms redness, heat, and swelling.

*Inflammation* has a threefold form: firstly, there is erethistic inflammation. In this form neither the venous nor the arterial system takes part, but the capillary system only. In extremely vascular structures, the capillary network is enlarged, the capacity increased, the organ reddened, the warmth exalted. In less vascular structures, new capillaries are formed. Secondly, there is synochal, or sthenic inflammation, in which all the preceding phenomena are more marked. The arteries themselves, as well as the capillaries, are implicated. The capillaries become arteries, and the arteries are dilated and their tunics thickened. Thirdly, there is the torpid or asthenic inflammation. In this form, venosity, or venousness, is the distinguishing characteristic. The veins are dilated and become varicose, and the capillaries change into veins, as in chronic ophthalmia, and the chronic inflammation of the mucous membrane in which polypi form. Inflammations may, however, be divided into arterial and venous; the former corresponding to the sthenic form, the latter to the asthenic.

*Course of disease.* Under this head Schönlein states the doctrines usually propounded by systematic writers as to the exacerbations and remissions of disease, tracing them, however, to telluric and lunar influences, albeit without clear and definite views as to the modus operandi of these influences. If we understand Schönlein aright, they are in fact more typical than real. Man is a microcosm, and just as the barometer and magnetic needle have their maxima and minima, so the pulse has a maximum and minimum, &c.

*Doctrine of critical terminations.* Schönlein adopts this to its full extent. The crisis may be general, and operate through the kidneys. When this crisis happens, a burning sensation is felt in the genital organs, and a feeling of dragging in the lumbar region and along the urethra experienced. With these there are joined a frequent desire to pass urine, a dry, rough skin, thirst, and sometimes an intermitting pulse. When the urine is critical, it must be secreted in due quantity; in the beginning, it exhibits a cloudy appearance near the surface (nubecula), and afterwards in the middle (suspensum), and lastly, a precipitate, or sediment, which is easily commingled, is reddish, and is somewhat raised in the middle. At the same time, the skin is moist and perspirable, or a copious sweat actually breaks out. When the crisis takes place through the skin, it is reddened, warm, and soft; the pulse becomes soft and small, the urine scanty; the perspiration must be general and clammy (kleberig); the skin must be warm afterwards, and the patient feel sensibly relieved. Sometimes there are other marks of a cutaneous crisis, as exanthems, and which must be considered as local crises. Of this kind are the vesicles which appear on the abdomen in typhus, and on the thorax and about the mouth and nostrils in peripneumonia.

*Critical hemorrhages* appear only in synochal diseases. They break out in various parts, but with reference to the organ affected and the peculiarities of the patient. Critical hemorrhages from the nose are most frequent in the young, and when the affected organ is situate above the diaphragm, particularly if the disease be synochal. The premonitory signs are redness and tension of the face, red watery eyes, spectral flashes, singing noises in the ears, itching of the nose, weight in the temples and headache, particularly of the back of the head. A discharge of a serous fluid often precedes the hemorrhage; the carotids beat violently, and the pulse is dicrotic. When the organ affected is below the diaphragm, the bleeding takes place from the nostril of that side in which the organ is situate: for example, in splenitis it is from the left nostril, in hepatitis from the right (?). Critical hemorrhages from the uterus are most usual in synochal diseases of organs situate above the diaphragm; those of the rectum occur only in adults in whom the seat of the disease is below the diaphragm.

All crises, except those operating through the skin and kidneys, are local, according to Schönlein's views; a proposition not only opposed to sound physiology, but to experience. A diarrhœa is often the indicant and outlet of a general critical effort, and so also is a bronchorrhœa.

*The terminations of disease* may be: 1. In complete restoration to health. 2, In imperfect restoration. The terminations of inflammation are: *a.* The formation of pus, which Schönlein considers to be the secretion from a newly-formed mucous membrane, constituted by nature for the relief of the organism. *b.* In hydropical effusion. This is an electric process (!) modified by natural laws. It takes place where serous membranes are in contact, and are in polaric tension with reference to each other. Their natural function to excrete an aqueous vapour is exalted in disease, and water itself is poured out, the action being analogous to that between the earth and the atmosphere, in which the vapours or clouds are precipitated in the form of rain or dew. *c.* Effusion of plastic lymph. *d.* Gangrene. *e.* Arthritis; the serous tissues becoming the seat of earthy deposits.

*Metastasis.* A disease on disappearing may be immediately replaced by another. This process takes place according to three principal laws. 1. In tissues which having a fundamental similarity of structures are related to each other, as the muscles and the muscular fibres of the heart, bronchi, intestines, or the peritoneum and the synovial membranes. 2. In organs constituting a system, as the stomach, liver, and spleen in the chylopoietic system. 3. In organs which are in direct polarity, as the liver, which being rather the positive or oxygen pole is opposed to the spleen, the hydrogen, or negative pole.

*General Therapeutics.* We do not pretend, be it understood by our readers, to give anything like a detailed view of the system of Schönlein. Eleven hundred pages of closely-printed German are not so easily reduced in our crucible. The foregoing paragraphs must therefore be considered as exhibiting the general nature of his pathological views. In the following, we intend in like manner to give a general view of his therapeutics.

The first step in therapeutics is the examination of the patient, which comprises two points of notice, the object and the method. The object includes the form and character of the disease. The form depends on the three circumstances already stated; the character depends on the mode in which the egoistic principle reacts against the hurtful agents, and may be erethistic, synochal, or torpid. If the disease be considered as a struggle between the egoistic and planetary principles, it may be, 1, vital—2, morbid.

The examination of a patient may be either genetic or analytical. The genetic traces the history of the patient, notes the sex, age, temperament, and other personal characteristics; the mode of origin of the disease, the cause as assigned by the patient, the previous state of health, &c. These are followed by a detailed inquiry into the condition as to structure and function of every organ, and system of organs.

The indications of treatment are founded on the anamnesis, the diagnosis, the prognosis, and the idea of the disease. From these arise two classes of indications, namely, indications of life, and indications of disease; the object of the former being to maintain the integrity of the organs, of the latter to remove the disease from the organism. The latter is subdivided into the indicatio causalis; indicatio curatoria, and indicatio urgens or palliativa.

The indications of treatment vary as the character of the disease varies. If it be erethistic, the reaction against the hurtful agent is usually not greater than is necessary to remove it from the system; here the expectant method is alone necessary. When the disease has the synochal type, the reaction is greater than necessary, and must be checked. This may be done by two methods: the one the antiphlogistic, applicable to the vascular system; the other the sedative, applicable to the nervous system. Bloodletting is the principal of the indications in the former method, narcotics are the chief of the sedative remedies. It is interesting to observe the transition from the pure bitters to the pure narcotics; simply by the former containing less carbon. Quassia in large doses will act as a narcotic. The first class of narcotics, represented by opium and the papaveraceæ, acts on the cerebral system; the second acts primarily on the ganglionic system, and only secondarily on the cerebral. Belladonna, hyoscyamus, lactuca, &c., belong to it, and are best given in powder or extract. The third class acts on the spinal system, the communicating link between the cerebral and ganglionic. Prussic acid is at the head of this class, which comprises all vegetables containing the acid, as the amygdala amara, prunus lauro-cerasus, &c. A fourth class comprises those vegetables which contain an active principle, as cicuta, nicotiana, &c., and which act on the nervous system in general. To the soothing class of remedies belong also all those in which carbon predominates over hydrogen, comprising also the mucilaginous and fatty compounds, as aloes.

The diseases characterized by torpor, require to be treated by the roborant and excitant method. The excitants are: 1. Ammonia, and its preparations, which act directly on the vascular system: containing an excess of hydrogen, it is the counterpart of the narcotics, in which carbon predominates. 2. Those which act on the cerebral system are compounds of hydrogen and include animal secretions, as musk, ambergris, the poison of serpents, alcohol, &c. Among the excitants of the spinal cord are nux vomica, rhus toxicodendron, but especially arsenic and its compounds. The excitants of the ganglionic system are the fetid gums, anemone pulsatilla, certain metals as copper and silver; and "animal magnetism, a neutral product of two indifferent things, of which the one acts as the brain, the other as the heart, while here the soul is requisite in action on the one hand, and the will on the other." A long list of excitants of the chylopoietic, cutaneous, serous, muscular, and sexual systems, with minor subdivisions, is given, all of which, with a more detailed exposition of the doctrine of crises we shall pass over—to notice,

*The natural system of Schönlein.* We know disease by its phenomena or symptoms only; these therefore must constitute the basis of a natural system of classification. They indicate either derangement of function or change of structure. In disease of the heart, for example, the pulse may be smaller, or intermittent, the heart changed as regards firmness, thickness, colour, &c. In inflammation of the eye, there is redness, a structural change; impaired vision, a functional change. In forming a natural system, as well as either diagnosis, or prognosis, it is essential that the true value of symptoms be noted, and the accidental distinguished from the essential. Schönlein thinks that great errors have been made by combining distinctive and non-essential characters. Changes in the form and appearance of organs are more important than a change of colour. The chemical composition of the urinary secretion is more important than its quantity in a given time. Every phenomenon or symptom of disease has an absolute and a relative value. Delirium is a symptom of absolute value; but there may be delirium from cerebral disease, and the delirium of intoxication, in which cases its value is relative. A knowledge of the

4298

circumstances which enable the physician to acquire a knowledge of morbid phenomena is also important to right classification. A disease may be noted and known at a glance; but it may also be distinguished only after putting together fragments of observation elaborated from the circumstances which prevent a clear perception of the disease as a whole.

*The rules laid down by Schönlein for estimating the value of characters in disease.* Functional derangement of the more important organs or structures takes precedence of that of the less important. Symptoms of vascular disturbance are of higher value than symptoms of disturbed renal function. A functional symptom is the more valuable in proportion to the number and importance of other phenomena with which it is connected, as the dread of water in hydrophobia. The more dignified the organ affected, the more important the symptoms referred to. Example; the heart takes precedence of the skin. Lastly, objective symptoms are to be preferred to subjective.

Among the circumstances which prevent a due estimate of the characters of disease are, firstly, the phenomena are never permanent, or in other words, the disease is continually undergoing a metamorphosis. The symptoms are changed, modify each other, or totally disappear, to be succeeded by new symptoms. Secondly, one disease runs into, or arises out of another. Thirdly, two or more diseases may be coincident, and then the phenomena complicate each other. Thus syphilis and gout may be combined in the same individual, and a peculiar affection of the bones may result, having the characteristics of both diseases. The correct combination of phenomena so as to characterize a disease truly, is a peculiar talent. The mineralogist, or botanist, has his subject before him as a whole; not so the physician, who has to make up a whole from a number of disjointed and disconnected parts. It is in doing this that the tact and skill of the physician is most displayed; and its successful practice (constituting skill in diagnosis,) depends often as much upon the innate powers as the experience of the practitioner. Experience and observation are, however, always requisite. The forms of disease, as described in systematic works, are often ideal, and not seen in practice. They are also the less available from a want of a critical arrangement and a technical terminology. A nosography should be correct, simple, and complete. Those phenomena should be grouped together, which have a physiological relation, and the functional changes should correspond to the structural.

Two modes of classification may be adopted, the analytical and the genetic. The genetic rises from the special to the general, the analytical, on the contrary, descends from the general to the special. He who would discover a system, must adopt the genetic method; he who would propound one, must adopt the analytic.

*The genetic method of classification.* The fundamental principle to start from is this; that there is no individual disease, but only individuals diseased. There is no actually existing pneumonia, but only individuals affected with pneumonia. Now all these diseased individuals, who under all varying circumstances of age, sex, temperament, present the same phenomena constitute a species of disease, and those essential phenomena characterize that species. For example, all persons suffering from traumatic pneumonia invariably exhibit the same phenomena of cough, difficult breathing, dull tone on percussion, and crepitating râle, whatever may be their individual condition or circumstances. Consequently, to determine any species, it is necessary to distinguish the essential from the accidental phenomena. The genus is determined after the same method by collocating species having characters in common. In inflammation of the lungs, for example, we have a series of phenomena which, although differing as regards the specific characters, have the common or genetic marks of cough, shortness of breath, &c. These general principles are also applicable to the determination of orders and classes, and are in fact the principles of classification adopted by taxonomists generally. We will now analytically follow our author through his nosology.

*The nosology of Schönlein.* The classes are three, and their characteristics are structural. There are three fundamental tissues : zoogen or animal tissue; blood (the vascular system) and marrow (the nervous system); The first class is the morphæ, (μορφη), consisting in changes of the zoogen ; the second hematoses; the third neuroses.

The class of morphæ comprises all abnormal developments, and all morbid changes in the form (μορφη), or nutrition of organs, and all surgical diseases. The families of this class are seven : namely,

| | |
|---|---|
| Dysmorphæ, | Arrests of development. |
| Theromorphæ, | Malformations. |
| Hypertrophia, | Hypertrophy. |
| Atrophia, | Atrophy. |
| Stenoses, | Contractions. |
| Ectopiæ, | Dislocations. |
| Vulnera, | Wounds. |

These families are subdivided into genera. Dysmorphæ includes, *a.* Hydrotrachia or spina bifida. *b.* Hydrocephalus chronicus. *c.* Cryptorchidismus. Theromorphæ has one family only, namely, atresia ani. If we understand Schönlein aright, these forms of abnormal development are mentioned only because others of this class are surgical; to us a reason altogether futile. We can see no characteristic difference between closure of the anus and congenital hernia of the bladder ; or between certain forms of hermaphrodism or split penis, and orchidismus, or non-descent of the testicle. Mention ought surely to have been made here of the precocious development of the sexual organs occasionally observed in both sexes, but particularly in females, in whom menstruation has taken place at various ages from eight months to seven or eight years, (referred by Schönlein for this reason to the class Hæmatoses,) with development of the mammæ and other cutaneous structures belonging to the sexual system.

The class of hypertrophied structures is divided into four groups, comprising the nervous, muscular, glandular, and horny structures. We subjoin a *precis* of this portion of Schönlein's nosology.

### Class I.—Hypertrophy.

*1st Group.* Hypertrophy of the nervous system.

Genus 1. Hypertrophy cerebri. G. 2. Hypertrophy of the peripheral nervous system.

*2d Group.* Hypertrophy of the muscular system.

Genus 1. Hypertrophia cordis. G. 2. H. sphincteris ani.

*3d Group.* Hypertrophy of the glands and glandular structures.
Genus 1. H. mammæ. G. 2. H. uteri. G. 3. H. ovarii. G. 4. H. prostatæ. G. 5. H. glandulæ thyroideæ. Struma.

The Atrophiæ are arranged as follows: Genus 1. Atrophy of the stomach and bowels. Marasmus. Sp. 1. Marasmus infantum. Sp. 2. M. juvenilis. Sp. 3. M. senilis.
Genus 2. Atrophy of the nervous system.
Sp. 1. Atrophy of the spinal chord. Tabes dorsalis. Sp. 2. Atrophy of the brain. Cretinismus. *a.* C. alpinus. *b.* C. campestris. *c.* C. senilis.
Genus 3. Atrophy of the genitals.
Sp. 1. Atrophia virilis. *a.* A. leprosa. *b.* A. acquisita. Sp. 2. Atrophia genitalis fœminea.

The class of Stenoses is divided into groups thus: 1. Stenosis of the organs of sense. (Some of these belong to the Dysmorphæ.) 2. Stenosis of the alimentary canal. 3. Of the respiratory organs. 4. Of the heart. 5. Of the urinary organs. 6. Of the vascular system. 7. Of the female genital organs. Some of these being surgical diseases are only noticed cursorily: the following are the genera in each group:

*2d Group.* Genus 1. Contraction of the œsophagus. *a.* Dysphagia sclerosa. *b.* Dysphagia cardiaca. *c.* Dysphagia lusoria.
Genus 2. Enterostenosis. Ileus, or passio iliaca.
Genus 3. Rectostenosis. Stricture of the rectum.

*3d Group.* Genus 1. Laryngostenosis.

*4th Group.* Genus 1. Cardiostenosis.

The Ectopiæ is entirely a surgical class, and comprises all forms of herniæ, dislocations, and prolapsus of organs. Schönlein places varices and ecchymoses in this class, as ectopiæ of the blood.

*The Hæmatoses* constitute the largest and most important class of diseases. Schönlein arranges them into eighteen families, some of which are made up of a most extraordinary medley of affections: hæmoptöe, apoplexy, and menorrhagia, for example, are in the same genus. Indeed the class as a whole exhibits an utter failure of the inventor to carry out his own adopted principles of classification.

The characters of hæmatoses are: 1. Chemical and physical changes in the constituents of the blood. 2. A change in the temperature of the body. 3. If the organ affected be a secreting organ, the secretion is changed, and as a product of disease is characteristic of the hæmatoses. In phlogoses, there is a change in the renal secretion, in hepatitis in the hepatic, &c. 4. There may be changes in the quantity as well as the quality of the blood. As a coup d'œil of the hæmatoses will be worth pages of verbal criticism we present the class in its families, tribes, and species.

CLASS II.—HÆMATOSIS.

FAMILY 1. Erythroses—plethora.
*a.* E. Vera (spissitudo sanguinis.) *b.* E. Neonatorum. *c.* Menstruatio precox.

FAMILY 2. Phlogoses—inflammations.

1*st Group.* Inflammations of the vascular system.

Genus 1. Arteritis. *a.* Acute general arteritis. *b.* Chronic general arteritis. *c.* Inflammation of the aorta. *d.* Inflammation of particular arteries.

Genus 2. Phlebitis. *a.* Phlebitis of veins of the upper extremities. *b.* Phlebitis of veins of the lower extremities. *c.* Phlegmatia alba dolens. *d.* Inflammation of the external jugular vein. *e.* Inflammation of the umbilical vein. *f.* Inflammation of the descending vena cava.

Genus 3. Carditis. Cardiac inflammation. *a.* Pericarditis. *b.* Carditis serosa. *c.* C. rheumatica. *d.* C. polyposa. *e.* C. arthritica. *f.* C. scorbutica.

Doubtful forms. *a.* C. syphilitica. *b.* Hydrargyria.

2*d Group.* Inflammation of the nervous system.

Genus 1. Encephalitis. *a.* E. traumatica. *b.* Meningitis. *c.* Arachnitis (acute and chronic.) *d.* Encephalitis vera, or phrenitis. *e.* E. insolationis. *f.* E. potatorum. Delirium tremens. *g.* Delirium traumaticum. *h.* Encephalomalacia—softening of the brain.

Genus 2. Inflammation of the spinal chord. *a.* Spinitis. *b.* Meningitis spinosa. *c.* Myelitis vera.

Genus 3. Neuritis. Inflammation of nerves.

*a.* Ischias postica.

3*d Group.* Inflammation of the organs of respiration.

1st Division. Inflammation of the mucous membrane.

Genus 1. Laryngitis. 2. Tracheitis. 3. Bronchitis.

2d Division. Inflammation of the cellular tissues.

*a.* Pneumonia traumatic, acute, chronic. *b.* Pleuro-pneumonia; bilious-rheumatic. *c.* Pneumonia venosa.

3d Division. Inflammation of the serous structures.

Genus 1. Pleuritis, acute and chronic.

4th Division. Inflammation of the glandular structures.

Genus 1. Inflammation of the thyroid body. 2. Of the thymus. 3. Of the bronchial glands.

4*th Group.* Inflammation of the chylopoietic viscera.

1st Division. Inflammation of the lining membranes. A. Above the diaphragm. B. Below the diaphragm.

Genus 1. Odonitis vera. 2. Glossitis. 3. Angina (five species.) 4. Œsophagitis. 5. Gastritis mucosa, serosa, venenata. 6. Enteritis serosa, mucosa (acute and chronic.) 7. Colonitis—simplex—venenata. 8. Proctitis (inflammation of the rectum.) 9. Dysenteria (four species.)

2d Division. Inflammation of the glandular structures of the chylopoietic system.

A. The salivary organs.

Genus 1. Parotitis (five species.) 2. Pancreatitis.

B. The biliary organs.

Genus 1. Hepatitis. *a.* Acute. *b.* Chronic (four varieties.) 2. Lienitis.

C. The mesenteric glands and peritoneum.

1. Peritonitis. *a.* Muscularis. *b.* Membranacea, acuta and chronica. *c.* Miasmatic; puerperal, four species, namely, erythritic, inflammatory, erysipelatous or gastro-bilious, and typhoid.

*5th Group.* Inflammation of the urinary apparatus.

Genus 1. Nephritis. *a.* Acuta. *b.* Calculosa. *c.* Chronica.

2. Cystitis. *a.* Acuta. *b.* Erysipelacea. *c.* Chronica. *d.* Muscularis.

*6th Group.* Inflammation of the sexual organs.

A. The female genitals.

Genus 1. Ovaritis. 2. Metritis. *a.* Acuta. *b.* Chronica. *c.* Deformans. *d.* Mucosa.

B. The male genitals.

Genus 1. Orchitis, four species.

*7th Group.* Inflammation of the locomotive organs.

Genus 1. Myitis. 2. Ostitis. 3. Arthritis. 4. Dermatitis.

FAMILY 3. Neurophlogoses.

*1st Group.* Neurophlogoses of the nervous system.

Genus 1. Hydrocephalus acutus. 2. Trismus neonatorum.

*2d Group.* Neurophlogoses of the chylopoietic system.

Genus 1. Stomacace. Gangrene of the mouth. 2. Angina gangrenosa. 3. Gastromalacia, softening or perforation of the stomach.

*3d Group.* Neurophlogoses of the respiratory organs.

Genus 1. Angina membranacea, croup. 2. Bronchitis maligna. *a.* Acuta. *b.* Catarrhus suffocativus. *c.* Consecutiva. 3. Gangrena pulmonum.

*4th Group.* Neurophlogoses of the sexual system.

Genus. Metritis septica.

*5th Group.* Neurophlogoses of the skin.

Genus. Anthrax. *a.* Carbuncle. *b.* A. contagiosa. Allied forms. 1. Pustula maligna. 2. Tinea infernalis.

FAMILY 4. The Typhi. The plague and yellow fever probably are intended to be introduced in this family, instead of the next, to which, by what appears to be a typographical error, they are referred.

Genus 1. T. cerebralis. 2. T. ganglionalis. 3. T. petechialis or exantheticus (four species.)

FAMILY 5. The Cyanoses.

Genus 1. Peliolis, or purpura. *a.* Hemorrhagica. *b.* Rheumatica. *c.* Senilis. 2. Scorbutus. 3. Cyanosis. *a.* Cardiaca. *b.* Pulmonalis. 4. Sclerosis. Induration of the cellular tissue. 5. Hemorrhaphilia Hereditary hemorrhagy. 6. Chlorosis, (allied to the latter is an abnormal formation of fat.)

FAMILY 6. The hemorrhages are divided into groups, including the cerebro-spinal axis, respiratory organs, &c.

Genus 1. Apoplexia cerebralis. 2. A. spinalis (two species.) 3. Epistaxis. 4. Hæmoptöe. 5. Pneumorrhagia. 6. Hæmatemesis. 6. Melena. 8. Proctorrhœa. 9. Mictus cruentum. 10. Metrorrhagia.

FAMILY 7.—*1st Group.* Catarrh of the respiratory organs.

Genus 1. C. simplex. 2. C. contagiosus, or influenza. 3. C. pulmonalis, or blennorrhea. 4. C. senilis, or moist asthma. 5. Emphysema. 6. Morbilli (four species.)

*2d Group.* Catarrh of the alimentary organs.

Genus 1. Gastro-ataxia. *a.* G. saburralis or indigestion. *b.* G. pituitosa. 2. Febris gastrica. Febris remittens. 3. Febris mucosa. 4. Hepatalgia. Hepatitis nervosa. 5. Gastrodynia biliosa. 6. Febris biliosa. Febris ardens, (three species.) 7. Cholera morbus. 8. Diarrhea, (seven species.) 9. Helminthiasis. 10. Aphthæ, (three species.)

*3d Group.* Catarrh of the urinary organs.

*4th Group.* Catarrh of the genital organs.

FAMILY 8. The Rheumatic Affections.

*1st Group.* R. of the voluntary muscles; containing 11 genera.

*2d Group.* R. of the involuntary muscles; 3 genera.

*3d Group.*

Genus 1. Febris miliaria. 2. Sudor anglicanus.

FAMILY 9. Erysipelaceæ, or erysipelatous diseases.

*1st Group.*

Genus 1. Febris erysipelacea. 2. Angina erysip. *a.* Simplex. *b.* Aphthosa. 2. Rose of the intestinal mucous membrane. 3. Rose of the genital organs.

*2d Group.* Rose of the skin.

A. Flat roses. Genus 1. Erysipelas (with 11 species and varieties.)
Genus 2. Febris scarlatina, (four species.)

B. Vesicular or elevated roses.

Genus 1. Urticaria, (five species.) 2. Zoster. 3. Variola, (five species.) 4. Rubeola. 5. Pemphigus.

FAMILY 10. Skin Diseases.

We shall only indicate the groups of this and the remaining families.

*1st Group.* Crypto-impetigines. 2. Acne. 3. Herpes. 4. Porrigines. 5. Psoræ.

FAMILY 11. The Scrofulous Diseases.

*1st Group.* Scrofula of the lymphatics. 2. S. of the bones. 3. S. of the mucous membranes.

FAMILY 12. Tuberculous Affections.

FAMILY 13. The Phthises.

*1st Group.* Phthisis of the respiratory organs. 2. P. of the digestive organs. 3. Of the urinary system. 4. The genital system. 5. The nervous system.

FAMILY 14. The colliquative diseases.

FAMILY 15. The dropsical diseases.

FAMILY 16. The Dyschymoses, comprising icterus, urodyalisis, and dysmenorrhea.

FAMILY 17. The Arthritic Diseases.

*1st Group.* Hemorrhoidal Diseases. *a.* Regular. *b.* Irregular. 2. Podagraform Diseases. *a.* Normal. *b.* Abnormal.

FAMILY 18. The Carcinomatous Diseases.

The class of neuroses comprises the numerous and varied forms of neuralgia, hysteria, and spasmodic and convulsive diseases. Venereal affections are comprised in one family, forming also a distinct class, and with these the volume closes.

On looking over this nosological arrangement, we find as many anomalies as in any other. As propounding a natural system, it seems to us altogether artificial. The leading characters of the hæmatoses, namely, a morbid condition of the vascular system and of the blood, with increase or diminution of temperature, are by no means exclusively characteristic of the diseases grouped together under that designation. In numerous forms of neuralgia, in hysteria, in various forms of asthma, as, for example, the asthma urinosum, these characters are most prominent.

The family of the phlogoses alone are a natural group. But what pathological relation is there between insolation and delirium tremens, in group 2 of the phlogoses, that there is not between insolation and puerperal convulsions in group 2 of the neuroses? or between ischias postica (sciatica) and meningitis in group 2 of the phlogoses, that there is not between sciatica and the neuralgiæ properly so called, placed in the somatic neuroses?

In our opinion, the exanthematic typhus placed among the typhoid fevers, is, in all its pathological characters, one of the exanthematous fevers, and much more nearly allied to scarlatina, variola, or morbilli, than to typhus cerebralis. While measles are placed amongst the catarrhal affections, with influenza, catarrhus senilis, blennorrhea, &c., scarlatina and variola are grouped amongst the erysipelatous, with erysipelatous angina, and rose of the intestinal mucous membrane, in the same genus as urticaria! This breaking up of the class of epidemic exanthemata, so well defined, and so closely allied to each other, is alone a fatal objection to the adoption of Schönlein's nosology in Britain. This is one of the many anomalies we might notice, but these we leave to be detected by our readers, having already provided them the means of forming a judgment.

*Special Pathology of Schönlein.* Such being the nosology, what is the pathology of Professor Schönlein? We had marked several passages for criticism and animadversion, in which our author states not only erroneous views, but erroneous facts. Take, for example, the following description of cryptorchidismus, or non-descent of the testicle:

"The patient, although at the age of puberty, is imperfectly developed. The body is elongated, the patient being tall, but he is weak, and in appearance childish. The muscles are weak, and not consolidated: the external genital organs diminutive; the penis does not enlarge, the scrotum is contracted, and contains often one testicle only. There is no beard; the voice is unchanged at puberty, is unmanly, or the 'break' is peculiar, mixed up with bass tones. The patient cannot learn to pronounce certain letters, as particularly the letter R. The mind is also affected; it is childish. The testicles remain altogether in the abdomen, or in the inguinal canal, where they form a perceptible tumour, which may be mistaken for hernia. The absence of the testes from the scrotum, the want of manly development, and the absence of all symptoms indicating incarceration of a portion of intestine, are sufficient for distinguishing the two." (Part I, p. 61.)

The symptoms of *atrophy,* or non-development of the testicles, are here detailed. The pathology of cryptorchidismus has been recently discussed in this Review, (vol. XIV, p. 61,) and all that need be repeated here is, that in the above description, Schönlein betrays not only a want of practical or clinical skill, but also of literary and physiological knowledge. A testicle, if fully developed, is just as efficient within as without the abdomen, and the patient will have a bass voice, a developed penis, and other marks of virility, and be quite able to pronounce the letter R, although they *both* be stuck fast in the groins, if so be that they are so large as to simulate a hernial tumour.

Again: In describing phlebitis, no mention is made whatever of a symptom which, in Great Britain, is considered almost pathognomonic; namely, the formation of abcesses in different parts of the body. Schönlein states that coagulated fibrin is often found in the inflamed vein, and beneath this a pus-like fluid; and this is all. We observe that the *causos* of Aretæus, or *febris ardens*, is described amongst the venous inflammations, as inflammation of the ascending vena cava. We might note other views entertained by Schönlein, as, for example, that scrofulous and tubercular disease are not identical; that tubercles are the result of suppressed secretions or excretions. Thus, he describes menstrual tubercles from suppressed menses, puerperal tubercles from suppressed lochiæ, tubercles from drinking cold water, from repulsion of exanthemata and impetiginous diseases, and from suppressed gout,—the lungs being affected in all cases. Schönlein also objects to the modern doctrine that phthisis is but the continuation and higher development of tubercular disease. It often is so, he argues, when the lungs are the seat of the disease, but hepatic, or gastric, or muscular phthisis, occurs without tubercles having preceded it. In phthisis, according to Schönlein, a morbidly-secreting surface is formed within the organ affected, which secretes a peculiar fluid, commonly termed pus, but which is by no means identical with that fluid. This morbid surface has a great resemblance to mucous membrane. Yet entero-phthisis is described as dependent on *ulcers* of the intestines, which not unfrequently perforate the intestines. Further, phthisis meseraica is simply the mesenteric disease of infants, and phthisis hepatica is neither more nor less than abscess of the liver. Then we have phthisis of the brain, of the ovaries, of the bladder, &c. Here is another instance in which Schönlein sets aside not only the nomenclature generally received by medical writers, but the first principles of taxonomy. Phthisis, as the term is now used, is applied to a disease of the lungs only, accompanied with hectic fever and atrophy. No systematic writer has applied the term to *tabes* generally, to hectic fever, to hepatic abscess, or to mesenteric disease. Schönlein makes phthisis synonymous with hectic fever and marasmus, and then contradicts, as if he had made a great discovery, the general opinion, that phthisis, in its ordinary form, is a higher development of tubercular disease. We certainly have read the arbitrary definitions and reckless contradictions displayed by Schönlein, with very great astonishment.

*The Clinical Medicine of Schönlein.* Dr. Güterbock having been a clinical student with Schönlein, and so having had an opportunity of hearing and appreciating his clinical discourses, felt certain that good service would be done to the medical commonwealth by the publication of them. That the reader of these clinical discourses may not mistake their object and spirit, Dr. Güterbock premises a few observations. He remarks, in the first place, that they are not intended for beginners; that they do not profess to afford detailed descriptions of disease, or medicinal formulæ, or rules for prescribing. Schönlein takes it for granted that his hearers are already acquainted with the theory of medicine. He wishes rather to form the ripe student into the scientific practical physician; to teach him how to observe; how to apply his five senses to the detection and comprehension of the phenomena of disease, and his understanding to the elaboration of a true pathology. Schönlein therefore rather sketches the outlines for his hearer to fill up, than draws a finished picture.

Having said so much by way of preface, and as an explanation due to Schönlein, we take for our criticism the first case in the volume, which is one marked as *Typhus abdominalis*.

"2d Nov. 1840. Christian Kämpfer, aged 19, a weaver's apprentice. He states that he has felt poorly and languid for the last four weeks, his feet being too weak to carry his body. He has often experienced vertigo. His sleep was light, and often broken by dreams, and he had an oppressive pain in the forehead. Eight or nine days ago, as nearly as he can remember, he experienced frequent rigors, followed by a continued heat. These data are important, as, previous to this period, was the stage of opportunity, and with it commenced the second seven-day period. We now observe three series of symptoms in the case:

"1 Nervous symptoms, as weariness, dulness of the head, dizziness, tottering gait, sleeplessness.

"2. Symptoms limited to the intestinal mucous membrane. The abdomen is soft, not painful on pressure, even if the cæcal region be strongly pressed. Three more watery stools have been passed during the last 24 hours. The tongue with a white fur was dry at its point yesterday evening.

"3. Symptoms of reaction. The fever is distinctly remittent, the remission occurring in the morning, the exacerbation in the evening. The pulse was 104 yesterday evening, to-day it is 84. The skin is harsh, dry, and in the evening hot. The urine is turbid, and deposits a slimy sediment, not pathognomonic.

"After a comparison of these three series of phenomena, there can be no doubt that the case is one of abdominal typhus in the second seven-day period.

"In modern times, we have heard of attempts to cut short this disease, to render it abortive from the first, or at least to alleviate the symptoms. This method is directly opposed to that which is founded on the principle that, after rigors have once set in, and all the symptoms of the disease are manifested, it must be allowed to run its course, through all its stages. The elder physicians (as Hildebrandt, Stoll, Richter,) have attempted to cut short the disease by emetics, and maintain that these have been the most effectual when gastric symptoms are manifest. I must distinctly express my disapproval of this means, which I have never found beneficial, even when ipecacuan only has been given. So far from cutting short or ameliorating the disease, I believe emetics have had an injurious effect, especially when tartar emetic has been combined with ipecacuan. At least, I found it so, in the case of some strong healthy nurses in the Julius hospital at Wurzburg, who were attacked with typhus fever after nausea, taking cold, &c. An emetic was administered to them, and the tongue became still more coated. The stools were rendered also more frequent, and death in some instances took place on the fourth day. The similarity between this disease and the acute exanthemata ought not to be overlooked. As in the latter the eruptions are the most marked where the skin is irritated, (as, for example, after bleeding in smallpox, the pocks are most numerous around the wound in the vein,) so after the irritation of an emetic on the intestinal mucous membrane, in the former, the eruption is likely to be more vivid. It is for similar reasons that simple saline aperients are so hurtful in this disease. Partly for these theoretical reasons, and partly from experience, I have come to the conclusion, that the use of emetics for cutting short the affection ought to be abandoned. A more valuable remedy for this purpose is that recently adopted, namely, calomel. To Autenrieth is due the merit of having first used it for this purpose; he administered it so early as 1806 and 1807, continuing its use until the peculiar green evacuations were induced. An objection may be thus raised at once to its use: 'How, after warning us against the administration of the innoxious neutral salts, can you recommend calomel?' It is true that calomel excites alvine evacuations, but they are not such as follow on stimulation of the intestinal mucous membrane. Further: it is allowed that calomel is useful when it excites its peculiar evacuations, which were formerly thought to contain the constituents of the bile, but, according to more modern researches, is altered colouring matter of the blood. The difference between the operation of calomel and of the neutral salts, is further shown by this, that, while the diarrhea increases with the latter, and the stools become more watery, they are more and more consistent with the continued use of calomel; so that at last, aperients (clysters) must be had recourse to. In what stage of the disease ought calomel to be administered? All observations agree that its administration must be limited to the first seven-day period, and the beginning of the second, and that the earlier it is administered, the more notable are its good effects. Its use is contra-indicated when phenomena referrible to the intestinal mucous membrane, and nervous system, as tenderness of the abdomen, dry tongue, and frequent pulse intervene. Given at a late period, it is injurious; it is most useful on or before the fourth day.

"The mildness of the symptoms in the present case leads to the conclusion, that this method of cutting short the disease should be adopted. Opinions vary as to the proper dose of calomel. Some recommend from three to four grains every two or three hours, until the characteristic evacuations are produced: another method (originating with the Tubingen school,) is to give the full dose of a scruple; then to omit it the next day, then on the third day repeat the dose, until the evacuations are less frequent. We have on former occasions followed the former method, let us now adopt the latter; and since calomel is apt to excite acidity in the stomach, we will combine it with eight grains of carbonate of magnesia.

"3d Nov. The patient took a scruple of calomel yesterday at two o'clock, and up to the present time only three characteristic motions have resulted, nevertheless watery motions continue. At first there was vomiting; no tormina. The exacerbation was very slight; the pulse was 96 per minute, and the patient slept for some hours during the night.

"This morning the pulse is only 80: the skin is still tense and drier. The urine deposits a slimy precipitate. Since the kidneys appear inclined to act, while the skin remains inert, to excite the latter, we will give the acetate of ammonia, and early to morrow the scruple dose of calomel shall be repeated.

"4th Nov. Yesterday there was no stool; the exacerbation was still more slight than on the day before (pulse 84,) and this slighter exacerbation was followed by a quieter sleep. To-day the patient feels much stronger, and his head is better, although there is still some intolerance of light.

At half-past five this morning, he took another dose of calomel; but up to this moment (and six hours later,) there have been neither evacuations nor molimina. . . . . . The abdomen is soft and without pain, the tongue moist, and its yellow coat coming off, the fever moderate as yesterday, the pulse yet strong. (p. 6.)

On the 5th Nov. it is reported that an alvine evacuation took place 14 hours after the calomel was taken, and shortly afterwards three more. The stools were of a brownish or green colour. The pulse was 75, as on the previous evening. Schönlein conceiving the critical day to be near, and the crisis in this disease occurring through the skin, warm bathing and diaphoretic drinks were ordered. On the 6th of Nov. the patient complained of weakness and sleepiness, and seemed more soporose. The warm bath was followed by a copious sweat, the skin becoming softer. The patient was quiet during the night, but it was rather a state of stupor than regular sleep. The head not better than usual. On the 6th Nov. the same soporose state continued; the tongue coated, but moist; little or no thirst; the abdomen soft, and "the characteristic noise in the cæcal region." The fever and pulse as before. To encourage diaphoresis the dose of liquor of acetate of ammonia was increased from ʒvj to ℥j, with ʒj of the tincture of valerian. In the evening the warm bath, and if the sopor continued, sinapisms to the calves of the legs. From the 9th to the 14th, all the cephalic symptoms were aggravated. On the 9th the patient lay on his back in a state of stupor, from which he could be readily roused, to relapse again immediately. Urine passed involuntarily; head hot; pulse accelerated; abdomen tender on pressure; hemorrhage from the nose and delirium during the night. Leeches and lotions to the head, and a clyster of liquor of acetate of lead and starch. The acetate of ammonia and valerian continued. On the 10th the patient was in the same condition, the pulse small, the speech stammering, the abdomen tender on pressure. Schönlein inquires, "is the congestion of the head consensual, proceeding from the ulceration of the intestinal mucous membrane? or is it the consequence of the fever? or is it idiopathic? The questions are not easily answered." Blisters to the legs were ordered; the saturnine clyster repeated, and infusion of cinchona with gum and oil. On the 11th, paralysis, dilated pupil, and profound apoplexy are reported, and death on the same day.

"*Post-mortem examination.* On the muscular and peritoneal covering of the small intestines there were found small bodies, varying in size from a millet to a pea, and filled with a cheesy substance. In the lower portion of the small intestine, not far from the cæcum, were a few small ulcers, the largest no bigger than a lentil; these were in the act of healing. In the large intestine, about four or five inches from ilio-cæcal valve there were a few similar ulcerations, but a little larger. The affection of the intestinal mucous membrane was much less than is usually noticed in this disease. There were a few miliary tubercles on the pleura pulmonalis. On removing the skull, the convolutions of the brain were found flattened, and the sulci of the pia mater contained a yellowish gelatinous lymph. This was in greatest quantity at the base of the skull, around the chiasma and infundibulum. The vessels were congested; the lateral ventricles, and particularly the left, filled and dilated with fluid, so that the fornix was stretched and appeared softened. Tubercles, the size of beans, were found in both hemispheres of the cerebellum."

Schönlein remarks on the the paucity of ulcers in the intestines. He thinks that no one could justly attribute the cerebral changes to the large doses of calomel, but on the contrary, the post-mortem appearances corroborate the views as to the utility of that remedy in abdominal typhus!

We have given this case at some length, that our readers may have a clear idea of Schönlein's clinical practice and instructions, and that we need not multiply quotations. For ourselves, we can aver that we have written it with surprise and astonishment. The elaborate "Pathology and Therapeutics" of the author had prepared us for a logical, rapid, yet effective examination of the patient; a clear and discriminating view of the symptoms; a separation of what was essential from what was accidental; an exposition of the causal relations of the symptoms: a well-reasoned and decided plan of treatment. Nothing, we need scarcely say, of this kind is in the case before us. The information as to the early history of the patient, his temperament, constitution, expression of countenance, colour of skin, condition of the pupils, of the respiration, and other points of importance to be observed, are all thought sufficiently described, as Dr. Scharlau observes in the seven words: "Christian Kämpfer, nineteen years old, weaver's apprentice." There is not the slightest indication whatever of the causes of the disease, unless the imagination seize upon the term, "weaver's apprentice," and survey Ch. Kämpfer wearily passing the shuttle to and fro in some dark damp cellar in Berlin, with typhus raging around, himself insufficiently fed, pale, languid, and anemious, previous to an attack of abdominal typhus. Again, the tongue is briefly described as being coated with a whitish fur: a description giving the reader no idea whatever of the true state of the tongue without again drawing on his imagination. Was it a thick coat, or a thin one? Was the tongue dotted with red points? Was it flat, or round, or jagged at the edges, or *tremulous?* On all these points Schönlein is silent.

In fact, the symptoms do not warrant the diagnosis at all as a case of abdominal typhus, taking even his own description of that disease, as given in his Pathology. Abdominal tenderness is one of his pathognomonic symptoms, yet here we have it expressly stated that there was *no* tenderness on pressure, and although we think that abdominal tenderness is in no respect pathognomonic, because we know, from our own experience, that there may be extensive ulceration of the intestinal mucous membrane, without any tenderness whatever, yet we would leave this unstated, and judge Schönlein by his own views. There are a few symptoms referrible to the head, the tongue is whitish, the pulse accelerated in the evening (as it is in all cases,) and scarcely accelerated in the morning, a little feverish heat of the skin, and it is pronounced a case of abdominal typhus.

We will say nothing about the treatment; our readers are well able to judge of that; but we do hope that the Germans will no longer blame English physicians for administering large doses of calomel. From three to four grains is three or four times a greater dose than we ordinarily administer, but when we read of scruple doses!—

Of what disease did the young weaver die? Evidently not of abdominal typhus, as maintained by Schönlein, but of subacute arachnitis, consequent on either hydatid or tubercular disease of the brain. We say *either* hydatid or tubercular disease, because it is impossible to place any reliance on the description of the post-mortem appearances. The tubercles the size of beans in the cerebellum may possibly have been masses of echinococci or acephalocyst hydatids: similar smaller masses may have existed in the cerebrum, lungs, &c., and have been easily overlooked by so careless an observer as Schönlein here shows himself to be. Surely something might have been said respecting the state of the pulmonary mucous membrane of the heart and large vessels, stomach, liver, spleen; of all, or each of which there is not the slightest mention.

Turning from a case of acute to one of chronic disease, we select case 17. This was an individual suffering from a series of symptoms of very common occurrence. In the month of November, 1840, a weaver, aged 34 years, complains that for the last nine months he has experienced pain in the chest, when going up stairs or running, dry cough, palpitation, shortness of breath, and lassitude. He has an appetite, but after eating he feels a sensation of fulness and dragging in the epigastrium. He says he works in a spacious room, and has always been healthy, with the exception, that about nine years ago, he had the itch for three weeks, which was cured by sulphur ointment; but subsequently he occasionally felt little pimples between the fingers when warm in bed, but which were never permanent. On a stethoscopic examination of the heart, signs of dilatation of the left ventricle, with hypertrophy, and valvular disease, were observed. The left lobe of the liver was found to be tumid, and the feet œdematous, particularly in the evening. The skin and bowels acted regularly, but the urine was scanty, and high-coloured. The case being diagnosed as one of cardiac disease, with partial enlargement of the liver and incipient dropsy, Schönlein next inquires, "what are the causes of this disease?" and the answer is, "we find in the anamnesis no other disease than the itch." Thereupon we have a dissertation on the nature of metapsoral diseases, of the existence of which Schönlein declares he has no doubt whatever. It is impossible to quarrel with a man's convictions, but we must be permitted to declare as firm a conviction on our part, that in this case a three weeks' attack of itch was not the cause of the disease. It is not improbable that rare ablution of the skin, and a neglect of personal cleanliness (so frequently coincident with scabies,) by impairing the cutaneous function, has occasionally induced renal disorder, and from this *fons et origo malorum*, other chronic diseases have arisen, which may thus have been mistaken for the legitimate sequelæ of scabies. We have noticed this case, however, rather to show the mode in which Schönlein hastily jumps to a conclusion in accordance with some preconceived hypothesis, rather than from a scientific and logical inquiry. The whole of the evidence from which the conclusion just stated is deduced is, we assure our readers, before them. There is positively no reference whatever to the condition of the kidneys, no inquiry as to the presence of albumen in the urine, nor in fact any suspicion of it.

In coming to the conclusion, that the three weeks' attack of the itch which occurred nine years previously to the examination of the patient, was the cause of the disease, Schönlein expressly observes, how important it is in the treatment of chronic diseases to know their causes. For this reason, he states why he came to the conclusion just mentioned: 1. The appearance of little papulæ between the fingers, after an attack of scabies, is, according to his experience, characteristic of a metapsoral disease. 2. To the objection, that so great an interval elapsed between the cause and the effect as nine years, he advances that diseases of the heart are insidious, and that in this case, the disease may have existed long before the patient became conscious of it. 3. No other cause to which the cardiac affection might be attributed could be detected: the man had never suffered from any rheumatic affection. We have just indicated a cause of the disease in the disordered renal function, but how could it be possible to learn the causes of the affection without due clinical inquiry? We have not a word as to the habits of the patient; whether he was cleanly or uncleanly, intemperate or sober. We are in ignorance too of the temperament and constitution of himself or his parents. Were the latter subject to rheumatic or arthritic diseases? Was the patient himself corpulent, or "pasty," or lean? Was his hair turning grey, his teeth firm or decayed, his eye dull and muddy, complexion florid or bilious? Had he had hemorrhoidal affections? These and similar inquiries are altogether omitted; but the itch was inquired after, and successfully, for the chances are rather in favour of a poor weaver, aged 34, catching the contagion some time or other during his 34 years of life; consequently, no other disease can be discovered, and ergo the itch nine years ago was the cause! Can there possibly be a more lame, more impotent, more ridiculously absurd conclusion than this? After five days' treatment, Schönlein at last discovers that his patient had had an attack of rheumatic ophthalmia, previously to the cardiac affection, that his occupation would predispose to hepatic disease, and that he had in fact, on closer inquiry, no aversion to spirituous liquors.

Our readers will ask how the patient was treated. Digitalis, acetate of potass, and taraxacum, were given with the effect of improving the urinary secretion, both in quality and quantity, and with manifest relief to the patient. When it was found, that he was addicted to drinking, the moxa was ordered to be applied about half an inch from the left nipple, and the wound to be kept open as an issue.

We here close our criticism. We need not sum up. The candidate for fame has had a fair reviewal; the facts are before the large and intelligent jury constituted by the readers of the British and Foreign Medical Review. Their decision will, we believe, accord with our own,

The print and paper of the large volume on Pathology are wretched; but we believe Professor Schönlein is not responsible for the publication of this volume, or of that containing his clinical Lectures, as we understand they are given to the public without his imprimatur.

---

